# L-Arginine Grafted Chitosan as Corrosion Inhibitor for Mild Steel Protection

**DOI:** 10.3390/polym15020398

**Published:** 2023-01-12

**Authors:** Sani Nazifi Dalhatu, Kolo Alhaji Modu, Auwal Adamu Mahmoud, Zakariyya Uba Zango, Abdullahi Bello Umar, Fahad Usman, John Ojur Dennis, Ahmed Alsadig, Khalid Hassan Ibnaouf, Osamah A. Aldaghri

**Affiliations:** 1 Department of Chemistry, Faculty of Science, Abubakar Tafawa Balewa University, Bauchi 740101, Nigeria; 2 Department of Chemistry, Faculty of Science, Kano University of Science and Technology, Wudil 713281, Nigeria; 3 Department of Chemistry, Faculty of Science, Al-Qalam University Katsina, Katsina 820101, Nigeria; 4 Department of Chemistry, Ahmadu Bello University, Zaria 810107, Nigeria; 5 Department of Physics, Al-Qalam University Katsina, Katsina 820101, Nigeria; 6 Department of Fundamental and Applied Science, Universiti Teknologi PETRONAS, Seri Iskandar 32610, Perak, Malaysia; 7 CNR Nanotec, University Campus Ecotekne, 73100 Lecce, LE, Italy; 8 Department of Physics, College of Science, Imam Mohammad Ibn Saud Islamic University (IMSIU), Riyadh 13318, Saudi Arabia

**Keywords:** chitosan, corrosion, inhibitor, L-arginine, mild steel

## Abstract

Corrosion prevention has been a global phenomenon, particularly in metallic and construction engineering. Most inhibitors are expensive and toxic. Therefore, developing nontoxic and cheap corrosion inhibitors has been a way forward. In this work, L-arginine was successfully grafted on chitosan by the thermal technique using a reflux condenser. This copolymer was characterized by Fourier-transform infrared spectroscopy (FTIR), thermogravimetric analysis (TGA), and X-ray diffraction (XRD). The corrosion inhibition performance of the composite polymer was tested on mild steel in 0.5M HCl by electrochemical methods. The potentiodynamic polarization (PDP) and electrochemical impedance spectroscopy (EIS) results were consistent. The inhibition efficiency at optimum concentration rose to 91.4%. The quantum chemical calculation parameters show good properties of the material as a corrosion inhibitor. The molecular structure of the inhibitor was subjected to density functional theory (DFT) to understand its theoretical properties, and the results confirmed the inhibition efficiency of the grafted polymer for corrosion prevention.

## 1. Introduction

Corrosion is defined as the degradation of materials caused by chemical or electrochemical attacks within the working environment [1]. It resulted in material losses and economic disadvantages for partial or total replacement of equipment and structures [2]. It is considered as an electrochemical reduction–oxidation (redox) reaction that occurs on the surface of metallic materials, prompting the release of electrons by the dissolution of metal and their successive transfer to another position on the surface, causing the hydrogen ions to be reduced and resulting in gradual deterioration and subsequent failure of the host material [3]. Corrosion has not only economic implications, but also social, including the safety and health of people, either working in industries or living in nearby towns. The petroleum industry is one of the sectors most affected by corrosion due to the presence of many corrosive substances in oil, which affects transportation of the petroleum products through pipelines [4]. Furthermore, corrosion represents a significant threat to storing radioactive wastes for safe disposal, and in medical implants as it causes blood poisoning [5]. Thus, it has been recognized as a global problem, affecting various aspects of human endeavors.

Various approaches have been put in place to combat the persistent problem, with corrosion inhibitors being one of the most cost-effective and practical methods of preventing metallic corrosion in various corrosive media [6]. Corrosion inhibitors are compounds used in low concentrations to slow down or stop the electrochemical process [1]. Most conventional corrosion inhibitors contain one or more heteroatoms such as nitrogen, oxygen, phosphorus, and sulfur, and the other functional groups that possess lone pairs of electrons (such as amino and hydroxyl) [7]. It occurs when partially filled d-orbital metal atoms, such as iron, are oxidized. The nonmetal is reduced by gaining the electron from the metal to form an oxide. In the presence of air or moisture, it deposits on the surface of the metal substances, creating a corrosive layer on the surface of the material. The presence of a corrosion inhibitor limits the corrosion reaction by altering the reaction mechanism of the corrosion process and keeping its rate to a minimum, thereby, preventing economic losses due to the corrosion [4]. The effectiveness of an inhibitor depends on the ability of the inhibitor to interact with a metal surface by forming bonds with the metal surface through electron transfer. Inhibitors usually are adsorbed on the metal surface by dislodging water molecules on the surface forming a compact barrier. The availability of nonbonded (lone pair) and p-electrons in inhibitor molecules facilitate the electron transfer from the inhibitor to the metal [4]. The inhibition efficiency of the inhibitor depends on the stability of the chelate formed. Therefore, it directly depends on the type and the nature of the substituents present in the inhibitor molecules [8].

**Figure 1:**
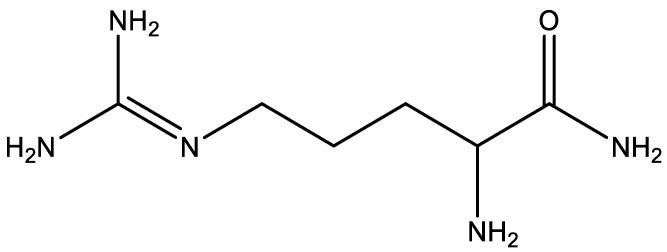
Molecular Structure of L-arginine (2-Amino-5-guanidinopentanoic acid).

The vast majority of conventional corrosion inhibitors are hazardous [9]. Thus, green corrosion inhibitors have been identified as the cheapest, most biodegradable, renewable, efficient, and ecologically friendly approach to reducing mild steel corrosion [10]. In recent years, there has been a rise in interest in using environmentally friendly, low-cost materials as corrosion inhibitors. This interest has grown to incorporate the use of polymers to prevent metallic corrosion [11]. Some researchers have claimed that chitosan is a suitable corrosion inhibitor for mild steel [6,12]. However, the extensive inter- and intramolecular hydrogen bonding of the polymer decreased its solubility in an aqueous and acidic environment, thereby lowering its inhibitory actions on mild steel [13,14]. Herein, we aim to boost the effectiveness of the polymer’s inhibitory actions by incorporating L-arginine (Figure 1). L-arginine is an amino acid (2-Amino-5-guanidinopentanoic acid) found naturally in proteins, in food such as seafood, watermelon, nuts, seeds, seaweed, pork, fish, and rich rice and soy proteins [15]. L-arginine itself has been reported as a corrosion inhibitor for mild steel by Khalid et al. [16]. Gowri and co-workers [17] reported the application of L-arginine derivatives as a corrosion inhibitor for steel in sea water.

Longer hydrocarbon chains in amino acids have often shown stronger corrosion mitigation [18]. As a result, adding more amino groups would enhance the electron density on the inhibitor molecule and, hence, the effectiveness of the inhibitor. Thus, the current research functionalized chitosan by grafting with L-arginine and utilized the copolymer as a mild steel corrosion inhibitor in 0.5M hydrochloric acid using electrochemical and density functional theory. The results show high inhibition efficiency even at a low concentration of inhibitor.

## 2. Materials and Methods

Chitosan of medium molecular weight (190–310 kDa and 75–85% degree of deacetylation), L-arginine, hydrochloric acid (HCl) and acetic acid were purchased from Sigma Aldrich (Shanghai, China). Tert-Butyl hydroperoxide, 70% solution in water was purchased from Thermo Fischer Scientific (Waltham, MA, USA). Other reagents such as acetone, ethanol was supplied by DKSH Specialty Chemicals (Bangkok, Thailand). All the chemicals used were of analytical grade and used without further purification. Mild steel coupons and emery paper were collected from the KhonKaen University workshop, Thailand.

### 2.1. Chitosan Modification

L-arginine was grafted on chitosan by dissolving 2 g of chitosan in 100 mL of 0.01 M acetic acid solution, and 2 g of L-arginine in 50 mL of acetone. These two solutions were mixed in a beaker and 1 mL of Tert-Butyl hydroperoxide (THB) was then poured into the resulting mixture. This mixture was then taken into reflux at 120 °C for two hours. Cs-g-L-arginine was then precipitated by adding 20 mL of 0.5 M NaOH to the mixture. The solution was filtered through 11μ filter paper. The filtrated Cs-g-L-arginine was then extracted with deionized water using Soxhlet extraction at 90 °C for six hours to remove excess homopolymer. The extracted copolymer was then dried in an oven at 60 °C for 6 h and stored in a desiccator (at room temperature).

### 2.2. Characterizations of the Copolymer

Fourier transformed infrared (FT-IR) spectra were obtained using FTIR infrared spectrophotometer model; Tensor27 S/N; 3683 (broker Hong Kong limited). Thermal analysis (TGA) was carried out using Hitachi TG/DTA thermogravimetric analyzer instrument model no. STA7200 Hitachi, using a scan rate of 10 °C/min range 25 °C to 600 °C, and X-ray diffraction (XRD) spectra were analyzed using Empyrean X-ray Diffractometer from Malvern.

### 2.3. Corrosion Analysis

The electrochemical test was carried out using a Metron auto lab Potentiostat (company) equipped with NOVA 1.1 software. Three electrode systems consist of a saturated calomel electrode as a reference electrode, mild steel with 1 cm^2^ exposed areas as a working electrode, and copper wire as a counter electrode. The open-circuit potential was set for one hour before any electrochemical measurement to maintain the steady-state potential. 

#### 2.3.1. Potentiodynamic Polarization (PDP)

The potentiodynamic polarization was carried out within the potential range of −8 V to +2 V using the scan rate of 0.001 Vs^−1^ and 303 K, the corrosion parameters were extrapolated from the Tafel plot fitting. The potentiodynamic corrosion inhibition efficiency (% *IE*) was calculated using Equation (1)

(1) $$\%IE_{P}=\frac{i_{corr}-i_{corr}^{\prime }}{i_{corr}}$$

where %*I E_p_* is potentiodynamic inhibition efficiency $i_{corr}$ and $i_{corr}^{\prime }$ was corrosion current density in the absence and presence of the inhibitor, respectively.

#### 2.3.2. Electrochemical Impedance Spectroscopy

The electrochemical impedance spectroscopy (*EIS*) was analyzed with the frequency range from 100 Hz to 10 MHz at 303 K. The electrochemical corrosion circuit was used to fit the corrosion data. The electrochemical corrosion inhibition efficiency % *IE_EIS_* was calculated by Equation (2)

(2) $$I\;E_{EIS}=\frac{R_{p}-R_{p}^{0}}{R_{p}}$$

where $R_{p}\;and\;R_{p}^{0}$ are resistance polarization values with and without inhibitor, respectively.

## 3. Results

### 3.1. FTIR and XRD Spectral Analysis

**Figure 2:**
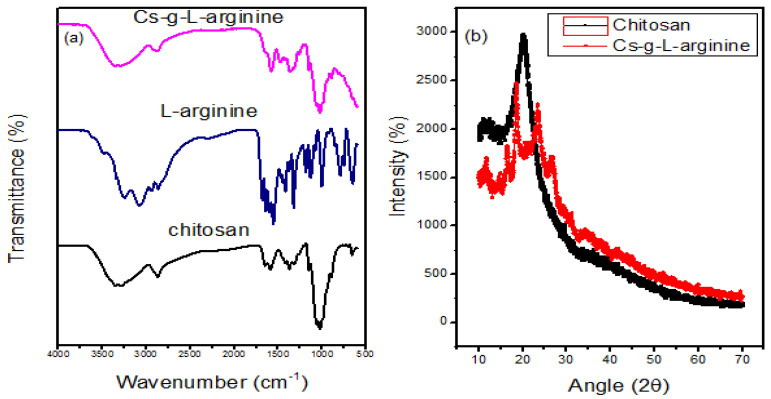
(**a**) Attenuated Fourier Transform Infrared Spectra of Chitosan, L-arginine, and Cs-g-L-arginine (**b**) X-ray Diffraction Spectrum.

Figure 2a displays the FTIR spectrum of pure chitosan, which has broadband at 3355 cm^−1^ and corresponds to stretching vibrations of O-H and N-H overlapping each other [18,19]. The stretching of the amide II bands correspond to 1603 cm^−1^ the band at 1591 cm^−1^ correspond to N-H bend [20]. The band at 1420 cm^−1^ correspond to symmetrical deformation of CH_2_ and CH_3_ [20]. The FTIR spectrum of Cs-g-L-arginine shows a C=O stretch at 1603 cm^−1^, the subsequent shift from 1603 cm^−1^ to 1685 cm^−1^ (in pure L-arginine spectrum) indicating that L-arginine was grafted on the chitosan [21]. The appearance of some peaks from the pure L-arginine spectra is evidence that the grafting process took place. The X-ray diffraction spectrum of Cs-g-L-arginine together with pure chitosan is presented in Figure 2b. The observed intense and strong broad peak at 2θ = 20° confirmed the semi-crystalline structure of pure chitosan, which was like the report by Thankamony et al. [22]. In comparison to pure chitosan, Cs-g-L-arginine had less intense and much broader peaks, indicating that grafting chitosan with L-arginine deformed the crystal zone in the chitosan system, making it less crystalline [15]. Furthermore, the formation of new peaks in the Cs-g-L-arginine spectrum is another indication that the chitosan L-arginine was grafted on chitosan.

### 3.2. Thermal Analysis

**Figure 3:**
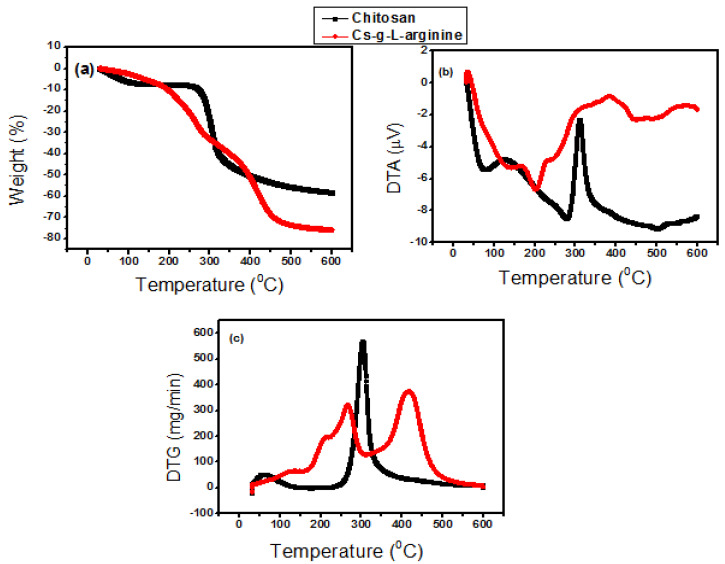
Thermal analysis: (**a**) Thermogravimetric analysis TGA, (**b**) Differential thermal analysis DTA, and (**c**) DTG of chitosan and Cs-g-L-arginine.

The TGA curve of chitosan in Figure 3a shows two-stage degradation. The first weight loss of 6.58% around 100 °C is attributed to the evaporation of water [20]. A sharp weight loss (51.47%) around 297–450 °C is due to the depolymerization of chitosan and the decomposition of the amine group [23,24]. On the other hand, the Cs-g-L-arginine thermogram shows a three-step degradation pattern, with the first weight loss (11.25%) around 110 °C, which is associated with loss of water. The second step is around 271 °C to 320 °C (40.34%), which is associated with the depolymerization of chitosan and the decomposition of L-arginine from the chitosan backbone [25]. A third degradation step was observed from 320 °C to 500 °C. DTA (Figure 3b) of pure Cs recorded in the air show a sharp exothermic peak around 300 °C, which is accompanied by thermal pyrolysis of the chitosan and thermal decomposition of amino and N-acetyl residues [23]. Similarly, the Cs-g-L-arginine curve shows a two less intense exothermic peak around 270 °C and 400 °C. The DTG of pure chitosan exhibited a maximum thermal decomposition temperature (T_max_) of 300 °C. However, Cs-g-L-arginine has a Tmax of around 400 °C. The DTG (Figure 3c) maxima temperature order is the same as that found in the DTA curve. The results from the thermal analysis indicate that grafting with L-arginine results in a drop in the thermal stability of the polymer [25]. Since the structure of pure chitosan has a significant amount of intermolecular hydrogen bonds, adding L-arginine will distort the hydrogen cluster in this structure, which results in the decreases of thermal stability of the chitosan.

### 3.3. Corrosion Analysis

#### 3.3.1. Potentiodynamic Polarization (PDP)

**Figure 4:**
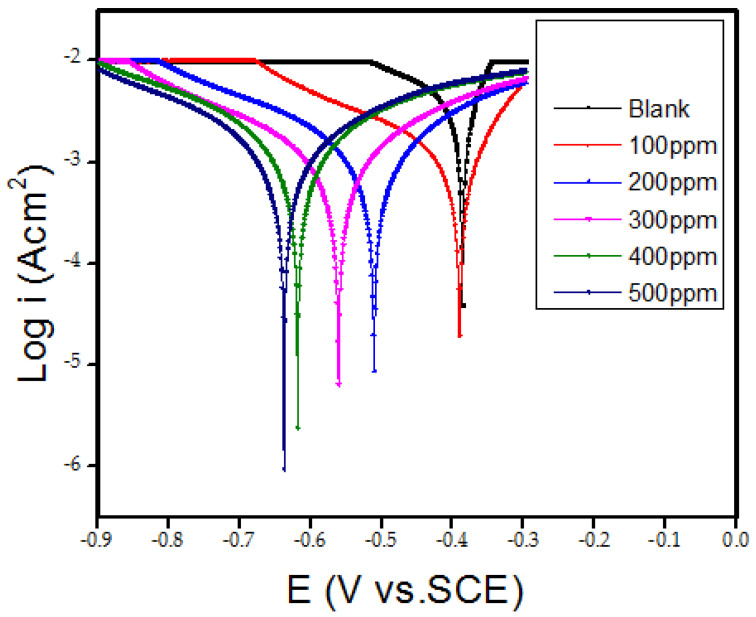
Potentiodynamic polarization curves for mild steel in the absence and presence of different concentrations of Cs-g-L-arginine (Tafel plot).

In Figure 4, Tafel plot shows the polarization curve of corrosion of mild steel in 0.5M HCl solution with different concentrations of the Cs-g-L-arginine. The electrochemical parameters such as corrosion rate, cathodic Tafel slope (βc), anodic Tafel slope (βa) values, corrosion potential (E_corr_), and corrosion current (I_corr_), were derived from Tafel plots by nova1.1 software. Tafel fit and percent inhibition efficiency (% *IE*) (calculated from polarization measurements according to Equation (1)) are shown in Table 1. From the Tafel plot, corrosion current density decreased with the addition of the inhibitor, due to the adsorption of the polymer to the surface of mild steel leading to decreases in the rate of dissolution of mild steel by blanketing the mild steel surface against the corrosive agent [26,27]. The cathodic Tafel slope (βc) values are less than that of the anodic Tafel slope (βa) values in most cases. This implies that the addition of an inhibitor demotes the iron (Fe) dissolution much higher than it retards the hydrogen evolution [12,28]. The inhibition efficiency increases linearly with increases in the concentration of Cs-g-L-arginine. At the optimum concentration of 500 ppm, the obtained inhibition efficiency was 91.4% with a little shift in the E_corr_ values towards the cathodic side. The corrosion inhibition mechanism is generally considered a cathodic inhibitor when the potential change exceeds 85 mV, while it is a mixed inhibitor when the potential change is less than 85 mV [29]. This indicates that Cs-g-L-arginine behaves as a mix type corrosion inhibitor. It is well observed that additions of the inhibitor are accompanied by lowering the corrosion current density related to the blank solution.

#### 3.3.2. EIS Analysis

**Figure 5:**
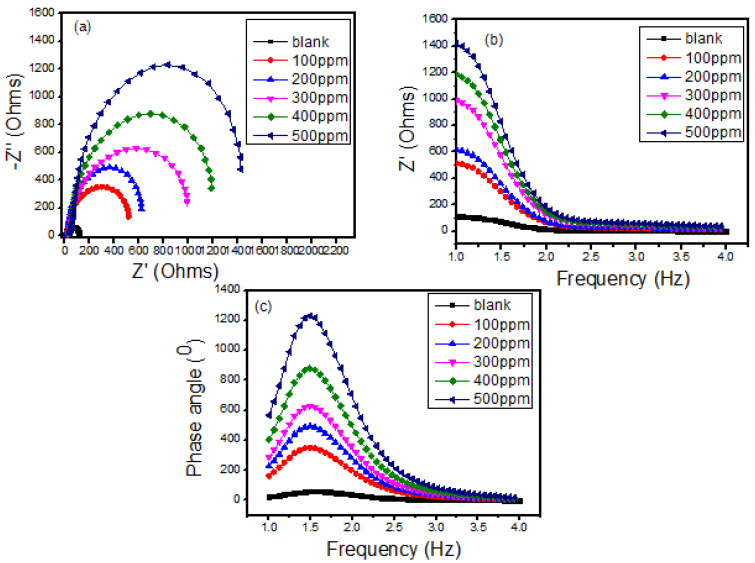
Electrochemical impedance Spectroscopy: (**a**) Nyquist Plot, (**b**) Bode plot, and (**c**) Phase angle plot.

The Nyquist plots for the corrosion of mild steel surface in 0.5 M HCl, inhibited by Cs-g-L-arginine, are depicted in Figure 5a the figures comprise a depressed semicircle; the presence of a capacitive loop in Nyquist curves is associated with charge transfer phenomena. The capacitive plots are semicircles, which can be attributed to frequency spreading caused by inhomogeneous electrode surface behavior [30]. The impedance response changed considerably after the addition of the inhibitor, i.e., an increase in the diameter of the Nyquist plots corresponds to an increase in inhibitor concentration. Shapes of the plots remained the same for the electrodes with and without various concentrations of inhibitors, indicating an unaltered mechanism of the corrosion process [28,29]. Figure 5b presented the Bode plot, in which log frequency is plotted against both the absolute values of the impedance (|Z|) and the phase-shift, which is one of the most used representation methods for electrochemical impedance spectroscopy results. The impedance values and phase angle values increase with the growing concentration of inhibitors. In addition, a time constant can be found in the phase angle, usually due to the relaxation effect of the corrosion inhibitor molecule adsorption [31]. The Bode plot (Figure 5b,c), in which log frequency is plotted against both the absolute values of the impedance (|Z|) and the phase-shift, is one of the most used representation methods for electrochemical impedance spectroscopy results. The impedance values and phase angle values increase with the growing concentration of inhibitors. In addition, a time constant can be found in the phase angle, usually due to the relaxation effect of the corrosion inhibitor molecule adsorption [32].

**Figure 6:**
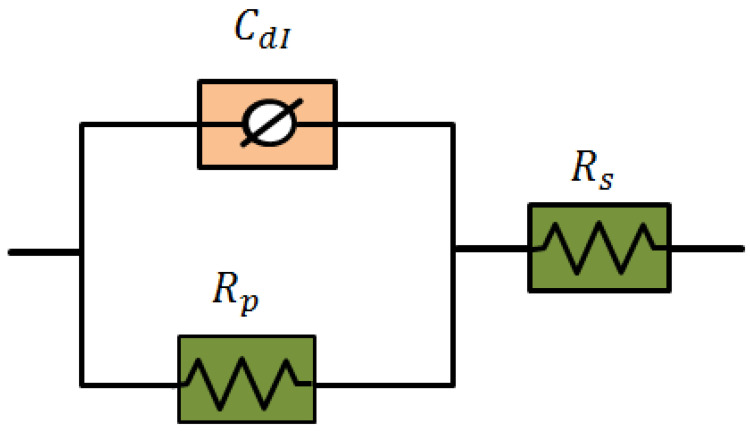
Equivalent circuit used to fit experimental data.

The electrochemical impedance parameters were obtained by fitting various impedance profiles into an equivalent circuit, which is given in Figure 6. This equivalent circuit is composed of constant phase element, CPE, solution resistance, Rs, and charge-transfer resistance, Rct. The system investigated here can be characterized by distributed capacitance for a nonhomogenous corroding surface of mild steel in 0.5 M HCl. This phenomenon of depression, modeled by CPE, is usually associated with the frequency dispersion, dislocations, surface roughness, formation of porous layers, and distribution of the active sites [28]. Table 2 shows that as the inhibitor concentration is increased, the Rct values increase, which can be attributed to the creation of an adsorption layer on the steel surface [33]. EIS findings revealed that the extent of corrosion inhibition by Cs-g-L-arginine has the highest corrosion inhibition efficiency of 91.4% at optimum conditions, indicating that the presence of an electron-donating group favors metal-inhibitor interactions. Furthermore, the Cdl values decreased, resulting in a higher concentration of inhibitor. This is caused by an increase in the thickness of the protective layer and/or a drop in the film’s local dielectric constant [31]. The good performance of the inhibitor is supported by a rise in charge transfer resistance values as well as the decrease in double-layer capacitance values obtained from impedance measurements. This behavior indicates that the inhibitors act as a barrier to the corrosion process, indicating the film’s formation [34]. By displacing H_2_O and other ions that were initially adsorbed at the steel/solution interface, the inhibitive layer on the electrode surface controlled the mild steel dissolution. In the presence of uninhibited 0.5M HCl, the Fe-H_2_O complex is generated [32]. This complex is now transformed into the Fe-Cs-g-L-arginine complex.

### 3.4. Quantum Chemical Calculations

#### Density Functional Theory (DFT)

**Figure 7:**
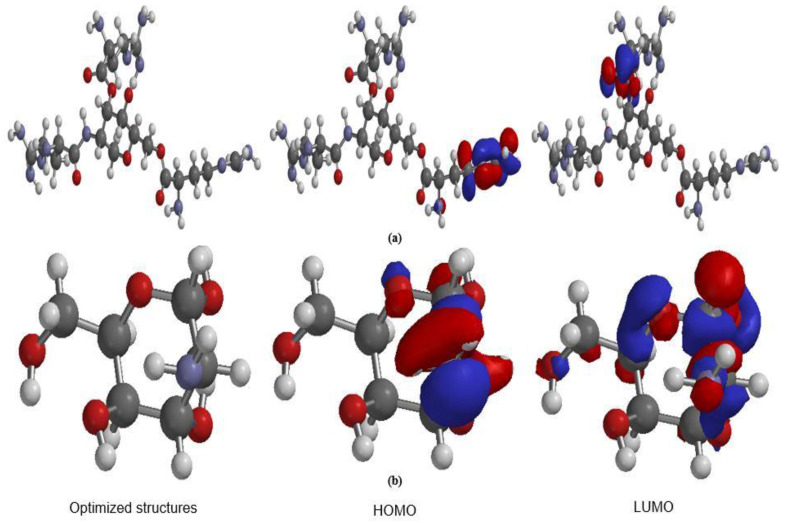
Optimized structures and frontier molecular orbitals electron density distribution (HOMO and LUMO) of: (**a**) Cs-g-L-arginine and (**b**) chitosan.

The effectiveness of an inhibitor is often determined by its structure and molecular orbital distribution. Quantum and theoretical chemistry have been known to be efficient tools for understanding corrosion inhibition mechanisms of the compound. Proper models with computational simulations based on quantum chemistry can support and confirm experimental discoveries. An inhibitor’s effectiveness is determined by both its spatial and molecular electronic structures. The optimized structure of chitosan-graft- L-arginine and pure chitosan are presented in Figure 7. As can be observed, HOMO is mainly localized over the L-arginine while, LUMO density is localized exclusively on the chitosan ring, indicating that these regions are mainly involved in electron(s) donation and acceptation, respectively, during the metal–inhibitors interactions [33].

Quantum chemical parameters are presented in Table 3. The smaller the value of ΔE, the more Fe interacted with the inhibitor, and hence, higher inhibition efficiency [35,36], the energy gap of Cs-g-L-arginine is much lower than that of pure chitosan, indicating that by grafting L-arginine on chitosan, one can increase the corrosion inhibition efficiency of the chitosan, this further supported the results obtained from electrochemical analysis. A high value of electronegativity of an inhibitor molecule shows the strong affinity of the molecule to accept the electrons from the metallic (Fe) surface. Furthermore, a molecule with higher electronegativity would have better interaction with the Fe surface and then better inhibition efficiency [36]. From the results, Cs-g-L-arginine has a higher value of X than that of pure chitosan; this indicates that the modified copolymer has better inhibition efficiency than that of pure chitosan. Hydrogen ions produced by the acid will turn to promote the anodic dissolution of the mild steel leading to various forms of corrosion [37]. Considering the nature of the inhibitor with respect to number of nitrogen atoms in the molecule, one may suggest that the inhibitor molecule will interact with the hydrogen ion and at the same time interact with the metal surface by donating the lone pair of electrons to the empty d –o and f- orbitals of the iron atom.

## 4. Conclusions

Since most corrosion inhibitors are hazardous and expensive, interest in using eco-friendly and affordable materials as corrosion inhibitors has increased significantly in recent years. This was expanded to include the utilization of natural substances to prevent metallic corrosion. Chitosan is a suitable option because of its desirable qualities; however, it was more effective at inhibiting growth when it was soluble in an acidic and aqueous environment. However, modifying chitosan with another molecule that can inhibit corrosion (such L-arginine) may increase the effectiveness of this inhibition. The L-arginine was successfully grafted on chitosan by thermal method; a modified copolymer was characterized by different characterization techniques. The corrosion inhibition efficiency of these polymers was tested on mild steel in 0.5 M hydrochloric acid by potentiodynamic polarization, electrochemical impedance spectroscopy and validated with DFT. The results show high inhibition efficiency by the modified polymer up to 91.4% at optimum conditions. The EIS analysis supported the inhibitory function of the co-polymer by demonstrating an increase in polarization resistance with increasing inhibitor concentration. A polarization analysis showed that the inhibitor molecule inhibits both cathodic and anodic corrosion processes, leading to its classification as a mix type corrosion inhibitor. The results of the inhibitor molecule’s quantum chemical calculations confirmed the findings of the experiment and offered proof of the interaction between the metal and the inhibitor. Hence, chitosan modified by L-arginine can serve as an alternative for corrosion mitigation methods.

## Figures and Tables

**Table 1:** Potentiodynamic polarization parameters.

Inhibitors	Inhibitor Concentration	$E_{corr}$ (mV)	$I_{corr}$ (mV)	(βa) (mV dec^−1^)	(βc) (mV dec^−1^)	*IE* %
Blank			70.997	175.65	174.69	
Cs-g-L-arginine						
	100	303.28	25.76	110.36	99.77	63.71678
	200	234.21	20.32	123.57	134.56	71.37907
	300	329.73	15.279	98.44	89.43	78.47937
	400	291.21	6.7083	80.45	70.76	90.55129
	500	305.9	6.05401	98.35	97.54	91.47287

βc= cathodic Tafel slope, βa = anodic Tafel slope, *E_corr_* = corrosion potential, and *I_corr_* = corrosion current.

**Table 2:** Electrochemical impedance spectroscopy parameters.

Inhibitor Concentration (ppm)	$R_{ct}$	N	$R_{s}$	Q	*IE* (%)
Blank	9.42	0.6998	2.1868	0.000448	
Cs-g-L-arginine					
100	28.86	0.62543	1.9875	0.005535	67.35967
200	32.654	0.66544	2.10443	0.004567	71.15208
300	40.765	0.71784	1.1794	0.001354	76.89194
400	54.334	0.64874	1.3688	0.002448	82.66279
500	109.98	0.65733	1.5188	0.002037	91.43481

*R_ct_* = charge-transfer resistance, *Rs* = solution resistance, constant phase element, N = CPE exponent Q = CPE constant.

**Table 3:** Quantum Chemical Parameters.

S/N		$E_{Homo}$ (eV)	$E_{Lumo}$ (eV)	ΔE	ɳ	σ (eV^−1^)	X	ΔN
1	Chitosan	−9.850	2.130	11.980	5.990	0.1669	3.860	9.404
2	Cs-g-L-arginine	−9.120	0.290	9.4100	4.705	0.213	4.415	6.081

ΔE = Energy band gap, ɳ = hardness, σ = softness, X = Electro negativity, and ΔN = fraction of electron transfer.

## Data Availability

The data will be made available on request.
